# Chronic viral infection impairs immune memory to a different pathogen

**DOI:** 10.1371/journal.ppat.1012113

**Published:** 2024-03-28

**Authors:** Cheng Yang, Zhicui Liu, Ying Yang, Luis J. Cocka, Yongguo Li, Weihong Zeng, Hao Shen

**Affiliations:** 1 Department of Infectious Diseases, the First Affiliated Hospital of Chongqing Medical University, Chongqing, China; 2 Department of Microbiology, University of Pennsylvania Perelman School of Medicine, Pennsylvania, Philadelphia, United States of America; 3 Department of Dermatology, Shanghai Tenth People’s Hospital, Tongji University School of Medicine, Shanghai, China; 4 Hainan Academy of Medical Sciences, Hainan Medical University, Hainan, China; 5 Shanghai Key Laboratory of Embryo Original Diseases, the International Peace Maternity & Child Health Hospital, Shanghai Jiao Tong University School of Medicine, Shanghai, China; Oregon Health Science University, UNITED STATES

## Abstract

Chronic viral infections cause T cell dysfunction in both animal models and human clinical settings, thereby affecting the ability of the host immune system to clear viral pathogens and develop proper virus-specific immune memory. However, the impact of chronic viral infections on the host’s immune memory to other pathogens has not been well described. In this study, we immunized mice with recombinant *Listeria monocytogenes* expressing OVA (Lm-OVA) to generate immunity to Lm and allow analysis of OVA-specific memory T (Tm) cells. We then infected these mice with lymphocytic choriomeningitis virus (LCMV) strain Cl-13 which establishes a chronic infection. We found that chronically infected mice were unable to protect against *Listeria* re-challenge. OVA-specific Tm cells showed a progressive loss in total numbers and in their ability to produce effector cytokines in the context of chronic LCMV infection. Unlike virus-specific T cells, OVA-specific Tm cells from chronically infected mice did not up-regulate the expression of inhibitory receptors, a hallmark feature of exhaustion in virus-specific T cells. Finally, OVA-specific Tm cells failed to mount a robust recall response after bacteria re-challenge both in the chronically infected and adoptively transferred naïve hosts. These results show that previously established bacteria-specific Tm cells become functionally impaired in the setting of an unrelated bystander chronic viral infection, which may contribute to poor immunity against other pathogens in the host with chronic viral infection.

## Author summary

The impact of chronic viral infections on protective immunity to other pathogens remains largely unknown. Our data showed that mice infected with a chronic virus were incapable of conferring protection against bacterial reinfection. Mechanistically, infection by chronic virus not only reduced the number of bacterial-specific memory T cells, but also led to dysfunction of these cells as evident by diminished production of effector molecules and poor recall responses. Our results provide novel insights into immune mechanisms underlying the increased susceptibility to other pathogens in chronic virus infected hosts.

## Introduction

CD8^+^ T cells are an important component of the immune system in controlling intracellular pathogens. After acute infection, naïve CD8^+^ T cells become activated, proliferate and differentiate into a large population of cytotoxic and effector T cells, which contribute to the clearance of pathogens [1]. Following antigen clearance, the expanded effector CD8^+^ T cell population contracts by apoptosis, with a minor subset, termed memory precursors, surviving and further differentiating into mature memory T (Tm) cells [2–4]. Tm cells acquire stem cell-like property of self-renewal in the absence of antigenic stimulation [5]. When re-encounter with antigen, these memory subsets are able to immediately produce a large amount of effector cytokines and rapidly proliferate into secondary effectors to provide strong protective immunity [6–9]. Therefore, proper maintenance of Tm cell populations is crucial to sustain long-lasting protection against re-infection by previously encountered pathogens.

In contrast to acute infections, antigen-specific T cells fail to differentiate into memory subsets during many chronic infections. Activated virus-specific T cells gradually lose the capacity to produce cytokines, such as IL-2, TNF-α and IFN-γ, and exhibit reduced cytotoxicity, poor memory recall responses [10]. They also show progressive loss in numbers due to impaired homeostatic proliferation [11]. This state of “exhaustion” is due to prolonged antigenic and inflammatory stimulation [12,13]. T cell exhaustion was first described in mice infected with the clone 13 (Cl-13) strain of LCMV [12]. Compromised T cell function has now been observed in other chronic viral infections both in animal models and humans, such as human immunodeficiency virus (HIV), hepatitis B virus (HBV) and hepatitis C virus (HCV) [14].

Chronic viral infections are common in humans, with each individual carrying 8~12 chronic infections on average [15]. Over the past two decades, tremendous efforts have been undertaken to uncover the molecular mechanisms underlying the exhaustion of T cells specific to the ongoing chronic viral infections, including the identification of transcriptional, epigenetic and metabolic alterations involved in driving this process [16]. However, little is known about how chronic viral infections may affect pre-existing bystander Tm cells and protective immunity to other pathogens that were induced by prior infections and/or vaccinations. This question is of great clinical relevance, since the functionality of the existing memory pool is critical to hosts’ immune competence, and any dysregulation to this memory repertoire may lead to increased susceptibility to re-infections from previously encountered pathogens [17].

In the current study, we infected mice with Lm-OVA to establish immunological memory to the bacteria, and subsequently infected these animals with LCMV Armstrong (Arm) or Cl-13 strain to establish acute or chronic infections, respectively. Our data showed that chronically infected hosts had significantly higher bacterial burdens and mortality rate following a bacteria challenge, and bacteria-specific Tm cells were functionally impaired. Specifically, we observed a large decrease in total numbers of bacteria-specific Tm cells, as well as their capacity to produce effector cytokines, leading to a failure in mounting a robust recall response by Tm cells both in the chronically infected and adoptively transferred naïve hosts. Unlike virus-specific T cells, which had sustained expression of inhibitory receptors during chronic viral infection, bacteria-specific Tm cells did not express high levels of inhibitory molecules (PD-1, LAG-3 and TIM-3). Our results provide direct evidence that competent adoptive immunity to previously encountered pathogens can be dysregulated by a bystander chronic viral infection, and uncover novel immunological mechanisms leading to impaired protective immunity to other pathogens in chronically infected hosts.

## Results

### Chronic viral infection impairs protective immunity to bacteria re-infection

**Fig 1:**
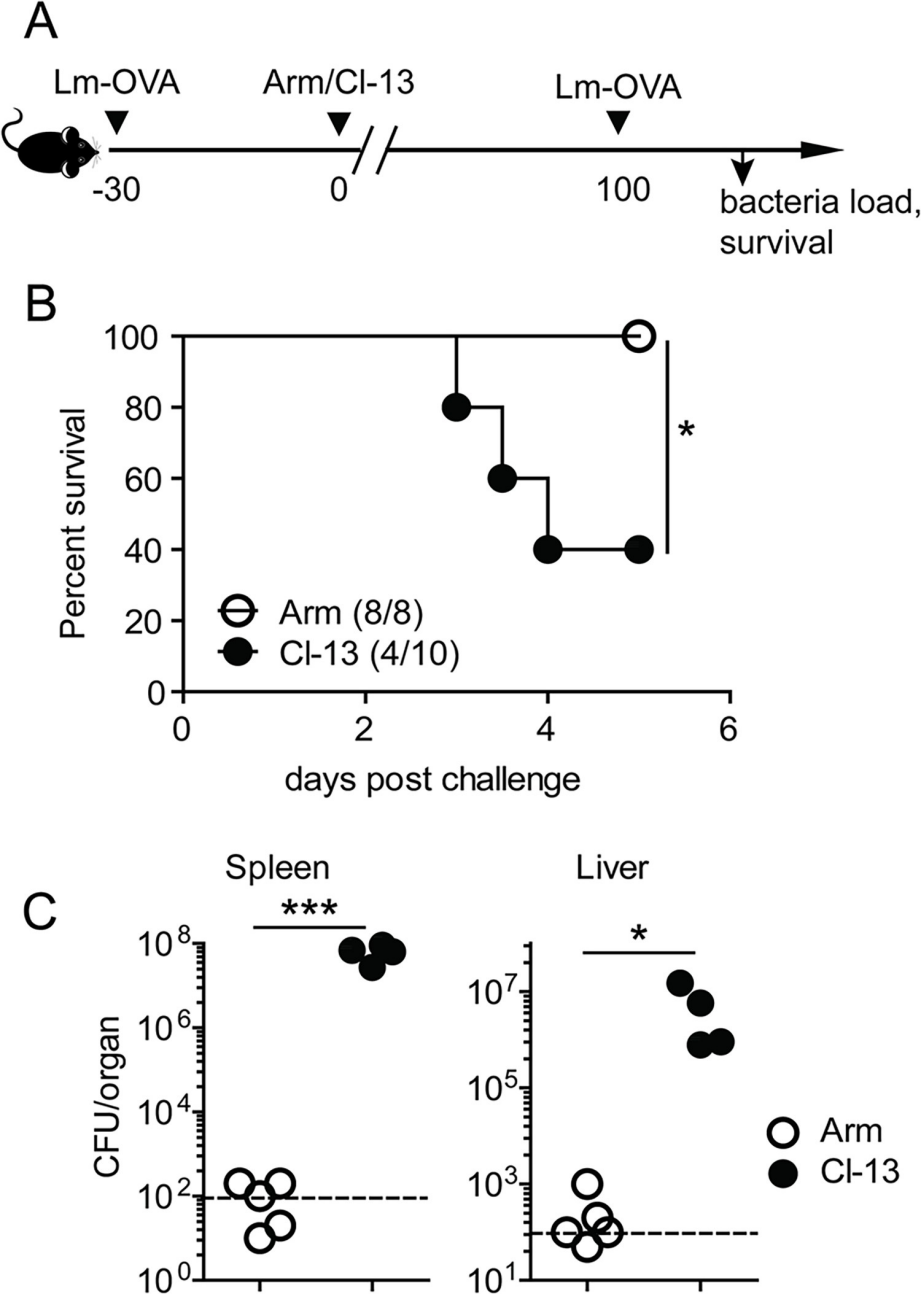
Chronic virus infection compromises protective immunity to Lm-OVA. (A) Experimental design to study the impact of chronic virus infection on protective immunity against Lm-OVA re-infection. Mice were immunized with Δ*actA* Lm-OVA (2×10^6^ CFU), and then infected with LCMV Arm (Lm-OVA/Arm) or Cl-13 (Lm-OVA/Cl-13) 30 days later. All mice were challenged with wild type (WT) Lm-OVA (1×10^6^ CFU) at day 100 after LCMV infection. (B) Survival of Lm-OVA/Arm and Lm-OVA/Cl-13 mice after Lm-OVA challenge. (C) Bacteria load in the spleens and livers of Lm-OVA/Arm or Lm-OVA/Cl-13 mice at day 3 after Lm-OVA challenge. * *p*<0.05; *** *p*<0.001.

To examine the effect of a chronic viral infection on protective immunity against an unrelated pathogen, we first immunized mice with recombinant Lm (Lm-OVA), which is a widely used model to study T cell mediated protective immunity [18]. More than 30 days after Lm-OVA immunization, we infected the immunized mice with either Arm or Cl-13 strain of LCMV that causes acute and chronic infection, respectively. As expected, LCMV-specific (D^b^/GP_33_^+^) CD8^+^ T cells from Cl-13 infected mice showed exhaustion phenotypes including: 1) higher levels of the inhibitory receptors (PD-1, TIM-3 and LAG-3), 2) downregulated expression of memory-associated molecules (CD44 and CD127), 3) less production of effector cytokines (IFN-γ, TNF-α and IL-2), and 4) reduced cytotoxicity as measured by expression of CD107a (a marker expressed on the cell surface of CD8^+^ T cells following stimulation with cognate peptides that correlates with cytolytic activity) [19] (S1 Fig). At day 100 post LCMV infection, we challenged these mice with a lethal dose of Lm-OVA (Fig 1A). All Lm-OVA-immune mice recovered from an acute Arm infection (Lm-OVA/Arm) were resistant to bacterial challenge, while more than half of Lm-OVA-immune mice with Cl-13 infection (Lm-OVA/Cl-13) died by day 5 post Lm-OVA challenge (Fig 1B). On day 3 after Lm-OVA challenge, the bacterial loads in the spleen and liver of Lm-OVA/Cl-13 mice were 4–6 logs higher than in those of Lm-OVA/Arm mice (Fig 1C). These results show that protective immunity against Lm re-infection is impaired by a chronic but not an acute LCMV infection.

### Chronic viral infection causes progressive loss of pre-existing bystander Tm cells specific to a different pathogen

**Fig 2:**
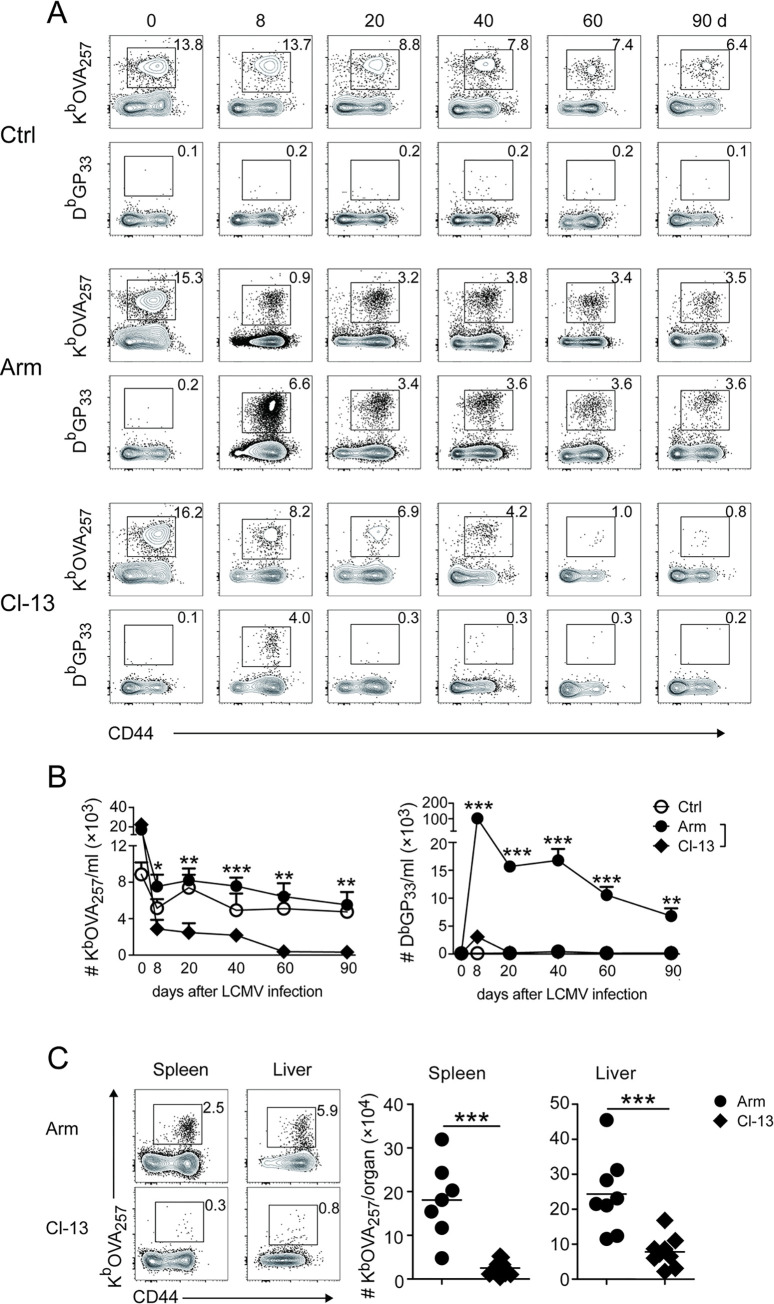
Chronic virus infection depletes Lm-OVA-specific Tm cells. Mice were immunized with Lm-OVA, and infected with LCMV as described in Fig 1. (A) Lm-OVA immunized mice were left uninfected (Ctrl) or infected with LCMV Arm or Cl-13, and representative figures of virus (D^b^/GP_33_)- or bacteria (K^b^/OVA_257_)-specific CD8^+^ T cells in the blood of these mice at different time points after LCMV infection were shown. (B) Quantification of K^b^/OVA_257_^+^ (left) and D^b^/GP_33_^+^ (right) CD8^+^ T cells in the blood of Ctrl, Arm and Cl-13 mice at different time points after LCMV infection. In order to better track dynamic changes of Lm-OVA specific T cells, mice with higher numbers of K^b^/OVA_257_-specific memory T cells were selected for infection with either Arm or Cl-13, while those with lower K^b^OVA_257_-specific memory T cells remained uninfected as Ctrl. Asterisks indicate the statistical significance between Arm and Cl-13 mice. (C) Representative dot plot (left) and numeric summary (right) of K^b^/OVA_257_-specific CD8^+^ T cells in the spleen and liver of Lm-OVA/Arm or Lm-OVA/Cl-13 mice at day 100 after LCMV infection. * *p*<0.05; ** *p*<0.01; ***p<0.001.

Chronic LCMV Cl-13 infection is known to induce functional exhaustion of LCMV-specific T cells [10]. To investigate possible mechanisms for the loss of protective immunity to Lm-OVA (Fig 1), we asked if pre-existing bystander Tm cells specific to Lm-OVA are also driven to exhaustion by chronic Cl-13 infection. Mice were immunized and boosted with Lm-OVA, and more than 30 days later were infected with LCMV Arm or Cl-13. The number, phenotype and functionality of both Lm-OVA-specific (K^b^/OVA_257_^+^) and LCMV-specific (D^b^/GP_33_^+^) CD8^+^ T cells were examined at different time points by serially bleeding and assayed by tetramers staining and intracellular cytokine staining (ICS). In Lm-OVA/Arm infected mice, while the frequency of K^b^/OVA_257_^+^ T cells decreased on day 8 following LCMV Arm infection due to extensive expansion of LCMV-specific T cells at this time point (Fig 2A), their numbers remained stable thereafter (Fig 2B). In contrast, the frequency and number of K^b^/OVA_257_^+^ Tm cells dropped progressively in Lm-OVA/Cl-13 infected mice, and by 60 days post Cl-13 infection, more than 90% of K^b^/OVA_257_^+^ Tm cells were depleted in the peripheral blood, resulting in a significantly lower number of K^b^/OVA_257_^+^ Tm cells in Lm-OVA/Cl-13 than Lm-OVA/Arm mice (Fig 2A and 2B). Notably, D^b^/GP_33_-specific T cells became almost undetectable in the blood of Lm-OVA/Cl-13 mice as early as 20 days after Cl-13 infection, which was much more rapid compared to the depletion of K^b^/OVA_257_-specific T cells (Fig 2A and 2B). The progressive loss of OVA_257_-specific CD8^+^ Tm cells was also evident when assayed by ICS for IFN-γ production following cognate peptide stimulation (S2A and S2B Fig). Similar results were observed in Lm-specific CD4^+^ Tm (LLO_190_^+^) cells, which were stably maintained in Lm-OVA/Arm mice but progressively lost in Lm-OVA/Cl-13 mice (S2C and S2D Fig). The profound loss of Tm cells was observed not only in the blood but also in the spleen and liver of Lm-OVA/Cl-13 mice, there were significantly lower numbers of K^b^/OVA_257_^+^ Tm cells in the organs of Lm-OVA/Cl-13 than in those of Lm-OVA/Arm mice on day 90 after LCMV infection (Fig 2C). Together, these data show that chronic viral infection leads to the progressive loss of pre-existing Tm cells specific to other unrelated pathogens.

### Chronic viral infection induces functional impairment of pre-existing bystander Tm cells

**Fig 3:**
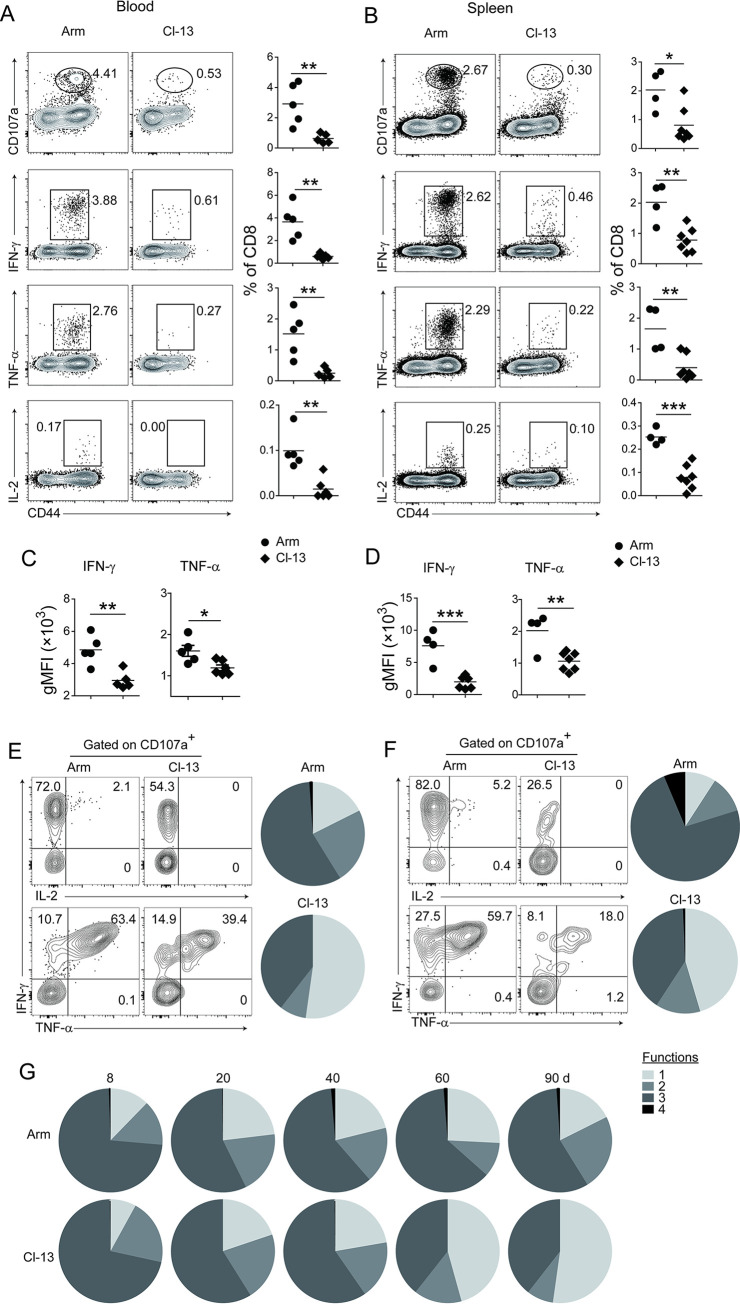
Chronic virus infection progressively impairs the function of Lm-OVA-specific Tm cells. Infection of mice was the same as described in Fig 2. (A and B) Percentages of CD107a, IFN-γ, TNF-α and IL-2 expression in CD8^+^ T cells after stimulation with OVA_257_ peptide in the blood (A) and spleen (B) of Lm-OVA/Arm and Lm-OVA/Cl-13 mice at day 90 post LCMV infection. (C and D) Per-cell levels of IFN-γ and TNF-α production in CD8^+^ T cells after stimulation with OVA_257_ peptide in the blood (C) and spleen (D). (E and F) Co-production of IFN-γ, TNF-α and IL-2 within CD107a^+^CD8^+^ T cells in the blood (E) and spleen (F) of Lm-OVA/Arm and Lm-OVA/Cl-13 mice at day 90 after LCMV infection, in the presence of OVA_257_ peptide stimulation. (G) Dynamic change of CD107a^+^CD8^+^ T cells that co-produce IFN-γ, TNF-α and IL-2 in the blood of Lm-OVA/Arm and Lm-OVA/Cl-13 mice, following OVA_257_ peptide stimulation. gMFI, geometric mean florescent intensity. Functions: 1, CD8^+^ T cells expressing CD107a alone; 2–4, CD8^+^ CD107a^+^ T cells co-producing one, two or all of the three cytokines (IFN-γ, TNF-α and IL-2).

In addition to profound loss of bystander Tm cells, the decrease of IFN-γ production indicated that functionality of bystander Tm cells might also be impaired by chronic Cl-13 infection. To further investigate this possibility, we determined the expression of CD107a and multiple cytokines (IFN-γ, TNF-α and IL-2) following OVA_257_ peptide stimulation of samples from blood, and spleens harvested at more than 90 days after LCMV infection. Compared with Lm-OVA/Arm mice, samples from Lm-OVA/Cl-13 group had much lower percentages of CD8^+^ T cells producing cytokines (IFN-γ, TNF-α and IL-2) or expressing CD107a (Fig 3A and 3B), indicating a reduction in cytokine production and cytotoxicity. Furthermore, OVA_257_-specific T cells from Lm-OVA/Cl-13 mice produced significantly lower amounts of IFN-γ and TNF-α on per cell basis as measured by geometric mean florescent intensity (gMFI) (Fig 3C and 3D). Since T cells producing more than one cytokine confer superior protection against invaded pathogens [20], we asked whether OVA_257_-specific T cells from Lm-OVA/Cl-13 mice were less polyfunctional in comparison with their counterparts from Lm-OVA/Arm mice. To this end, CD107a^+^ OVA_257_-specific CD8^+^ Tm cells were further analyzed for cytokines production at day 90 post LCMV infection. Most CD107a^+^ OVA_257_-specific CD8^+^ Tm cells co-produced at least two of the three cytokines (IFN-γ, TNF-α and IL-2) in Lm-OVA/Arm mice. By contrast, approximately half of them from Lm-OVA/Cl-13 mice produced none of the three aforementioned cytokines in the blood and spleen (Fig 3E and 3F). When analyzing the polyfunctionality of OVA_257_-specific CD8^+^ Tm cells at different time points after LCMV infection, we found that polyfunctional OVA_257_-specific CD8^+^ Tm cells remained stable in Lm-OVA/Arm mice until the end of our experiments. In Lm-OVA/Cl-13 mice, however, OVA_257_-specific CD8^+^ Tm cells lost the capacity to produce IL-2 as early as day 40 post Cl-13 infection. The proportion of multi-functional T cells decreased while monofunctional T cells (CD107a^+^/IFN-γ^-^/TNF-α^-^/IL-2^-^) gradually increased from day 60 after Cl-13 infection (Fig 3G). Similar to OVA_257_-specific CD8^+^ Tm cells, LLO_190_-specific CD4^+^ Tm cells also showed reduced polyfunctionality in Lm-OVA/Cl-13 mice (S3 Fig). Together, these data indicate that chronic viral infection not only causes progressive loss of pre-existing bystander Tm cells, but also impairs their effector functions.

### The majority of T cell population become partially dysfunctional after chronic viral infection

**Fig 4:**
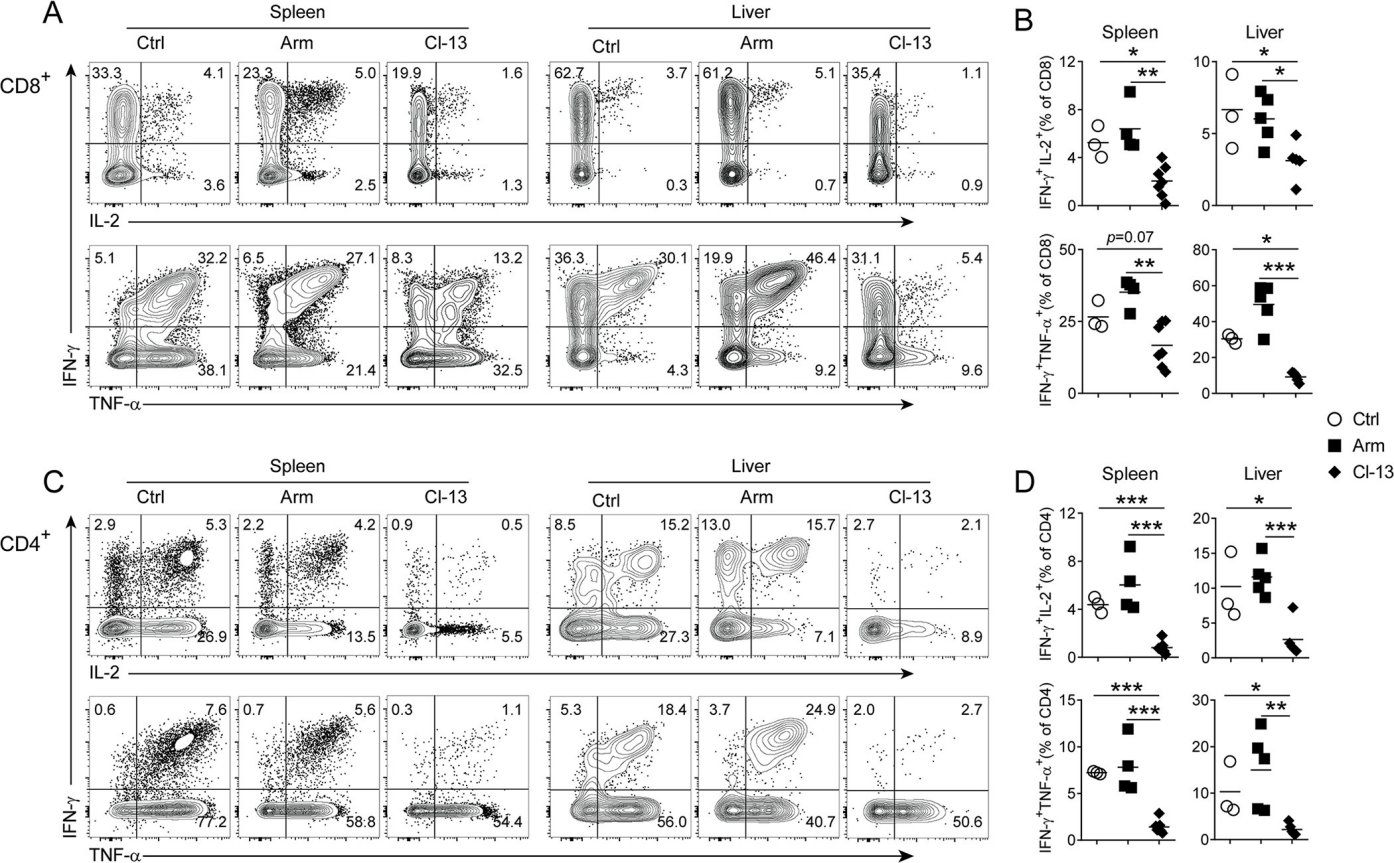
Chronic virus infection induces functional defects to the majority of T cell population. Infection of mice was mentioned in Fig 2. More than 90 days after LCMV infection, mononuclear cells isolated from the spleen and liver of Ctrl, Lm-OVA/Arm (Arm) or Lm-OVA/Cl-13 (Cl-13) mice were stimulated with PMA and ionomycin, followed by ICS for IFN-γ, TNF-α and IL-2. (A) Representative figures showing the production of cytokines by CD8^+^ T cells. (B) Statistical presentation of CD8^+^ T cells co-producing IFN-γ/IL-2 or IFN-γ/TNF-α. (C) Representative images for the production of cytokines by CD4^+^ T cells. (D) Summary graphs for the frequency of CD4^+^ T cells that co-produce IFN-γ/IL-2 or IFN-γ/TNF-α.

Since we have observed that both virus- and bacteria-specific T cells were functionally impaired after a chronic viral infection, we hypothesized that chronic LCMV infection may have broad impacts on the functionality of T cells. To test our hypothesis, we isolated mononuclear cells from the spleens and livers of Lm-OVA/Arm and Lm-OVA/Cl-13 mice at more than 90 days after LCMV infection, as well as Lm-OVA immune mice (no LCMV infection) as the control (Ctrl). We then performed ICS for multiple cytokines (IFN-γ, TNF-α and IL-2) following short stimulation with phorbol 12-myristate 13-acetate and ionomycin (PMA/I), which bypasses proximal TCR signaling and induce most effector and memory (but not naïve) T cells to produce cytokines [21]. The results showed that Ctrl and Lm-OVA/Arm group had similar numbers of CD8^+^ T cells producing IFN-γ, TNF-α and IL-2. In contrast, frequencies of CD8^+^ T cells producing IFN-γ, TNF-α or IL-2 were much lower in Lm-OVA/Cl-13 mice (Fig 4A). More importantly, a striking decrease was observed in the percentages of multi-functional CD8^+^ T cells co-producing IFN-γ/IL-2 or IFN-γ/TNF-α in Lm-OVA/Cl-13 mice, in comparison with Ctrl and Lm-OVA/Arm mice (Fig 4A and 4B). Similar results were observed in CD4^+^ T cells (Fig 4C and 4D). These data suggest that a large fraction of T cells, both virus-specific and non-virus-specific, are functionally impaired in the context of a chronic viral infection.

### Effects of chronic LCMV infection on bystander Tm cells differ from exhaustion of LCMV-specific T cells

**Fig 5:**
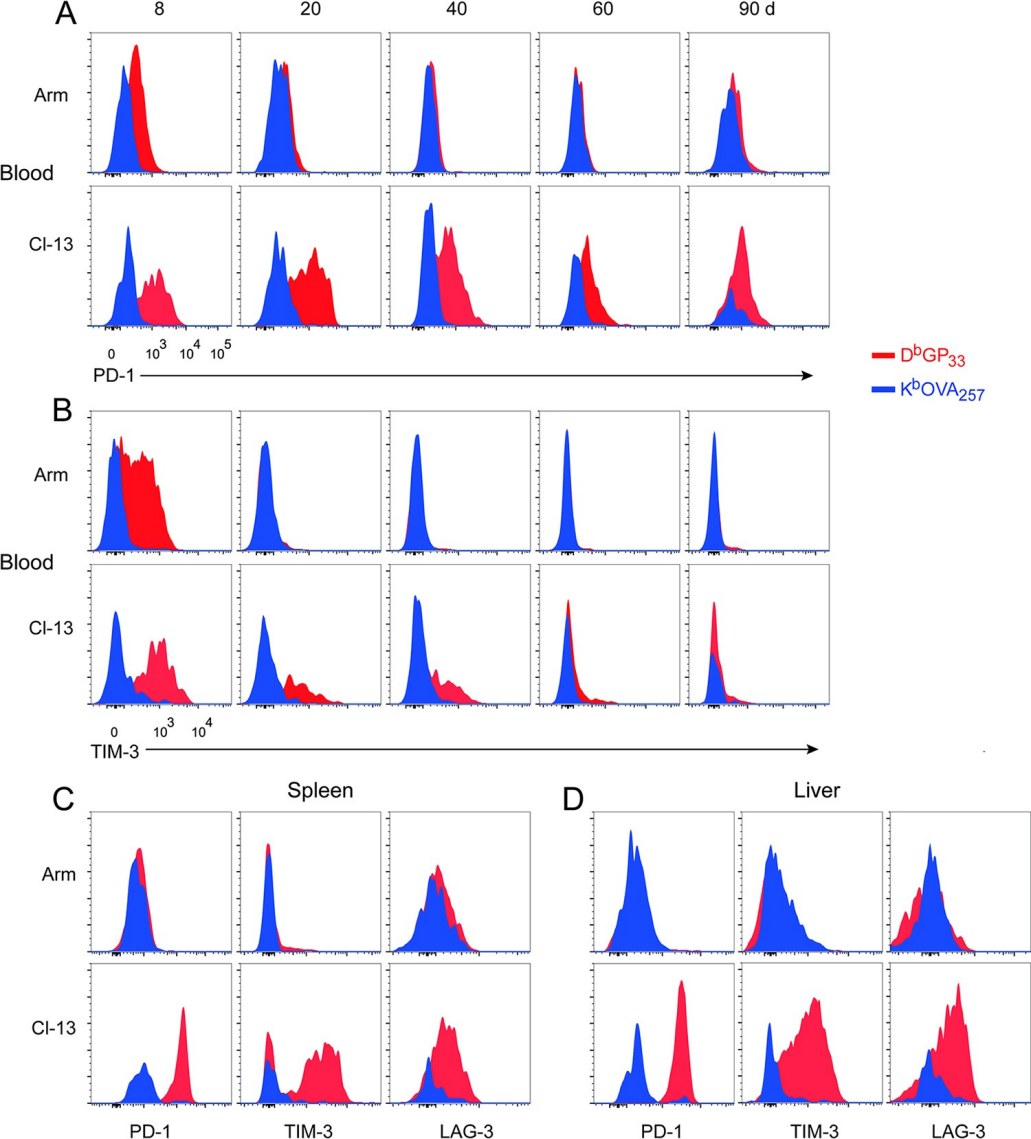
Lm-OVA-specific Tm cells do not up-regulate the expression of inhibitory molecules during chronic virus infection. Infection of mice was described in Fig 2. (A and B) Expression of PD-1 (A) and TIM-3 (B) on K^b^/OVA_257_- and D^b^/GP_33_-specific CD8^+^ T cells in the blood of Lm-OVA/Arm and Lm-OVA/Cl-13 mice at different time points after LCMV infection. (C and D) Expression of PD-1, TIM-3 and LAG-3 on K^b^/OVA_257_- and D^b^/GP_33_-specific CD8^+^ T cells in the spleens (C) and livers (D) of Lm-OVA/Arm and Lm-OVA/Cl-13 mice at day 90 after LCMV infection.

Loss of bystander K^b^/OVA_257_^+^ Tm cells was a slow process, becoming profound only after 60 days post Cl-13 infection. This was in contrast to the depletion of LCMV-specific D^b^/GP_33_^+^ T cells, which was evident as early as day 20 after Cl-13 infection (Fig 2). High and sustained expression of multiple inhibitory receptors is one of the major hallmarks of exhausted T cells [22,23]. To investigate whether bystander K^b^/OVA_257_^+^ Tm cells have the properties of exhausted T cells, expression of inhibitory receptors PD-1, TIM-3 and LAG-3 was monitored in Lm-OVA/Arm and Lm-OVA/Cl-13 mice. Consistent with previous reports [22,24], virus-specific T cells primed by Arm infection rapidly down-regulated the expression of PD-1. In contrast, expression of PD-1 was maintained at a high level for weeks post Cl-13 infection (Fig 5A). Interestingly, PD-1 was not up-regulated on bacteria-specific T cells at various time points post LCMV infection in the peripheral blood, regardless of Arm or Cl-13 infection (Fig 5A). A similar trend was also observed for the inhibitory marker TIM-3 (Fig 5B). At the end point of our experiments (> day 90 post LCMV infection), we measured the expression of inhibitory receptors on virus- and bacteria-specific T cells both in spleens and livers. We found that virus-specific T cells in Cl-13 infected mice expressed high levels of PD-1, TIM-3 and LAG-3 as expected. Unlike virus-specific T cells, increased expression of the three aforementioned inhibitory receptors were not observed on K^b^/OVA_257_^+^ Tm cells from Cl-13 infected mice (Fig 5C and 5D). Therefore, dysfunctional bacteria-specific Tm cells do not appear to display the major characteristics of T-cell exhaustion during a chronic viral infection.

### Bystander Tm cells derived from chronic virus infected hosts fail to recall upon bacteria re-challenge

**Fig 6:**
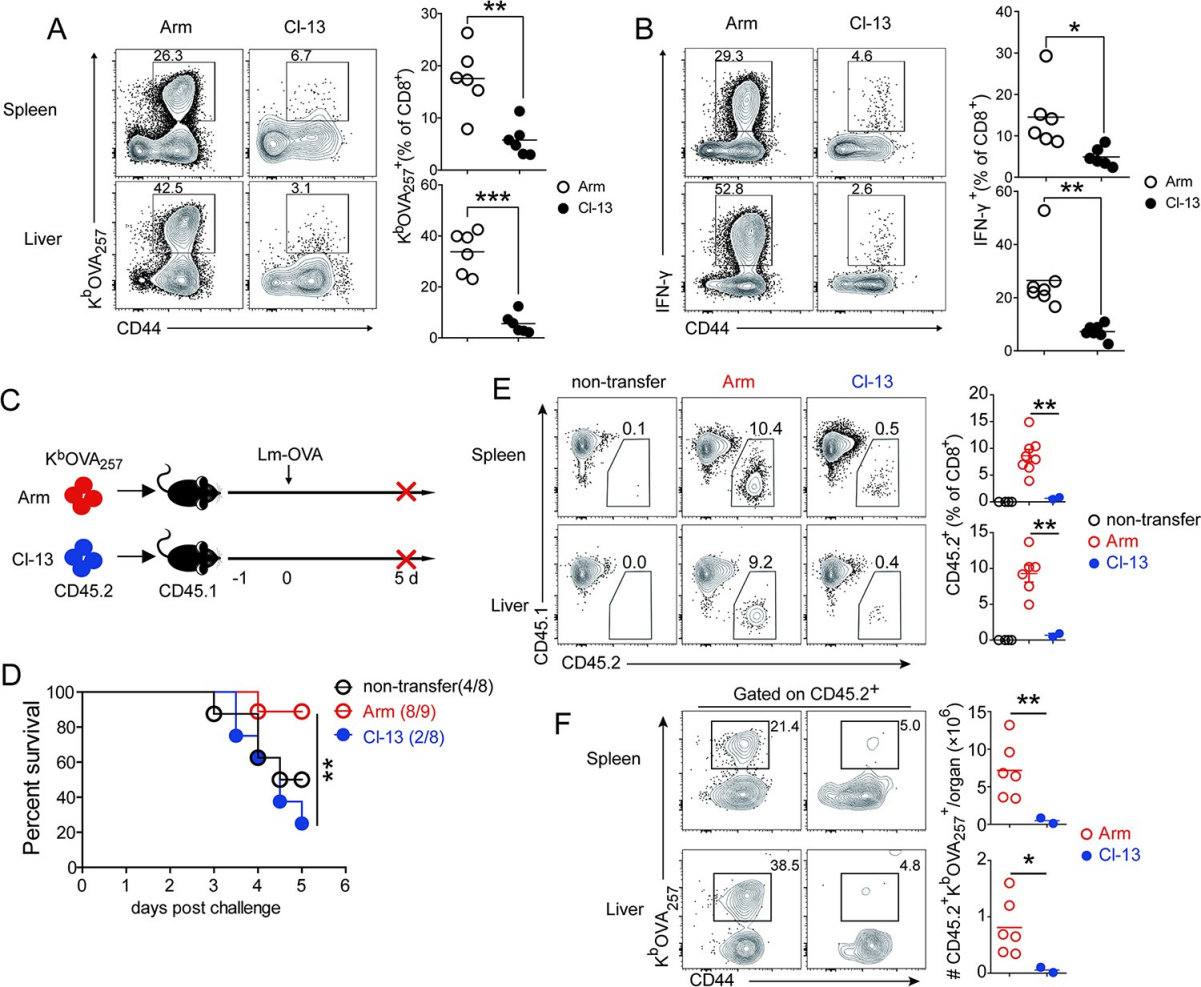
Chronic virus infection negatively impacts the memory recall of Lm-OVA-specific Tm cells. (A and B) Infection of mice was described in Fig 2. Mice were challenged with WT Lm-OVA on more than 100 days after LCMV infection, recall responses of K^b^/OVA_257_-specific Tm cells in the spleen and liver of Lm-OVA/Arm and Lm-OVA/Cl-13 mice were determined by MHC Class I tetramer staining (A) or ICS for IFN-γ after cognate peptide stimulation (B) on day 5 post Lm-OVA challenge. (C) Design for adoptive transfer experiment. CD8^+^ T cells were purified from the spleens of Lm-OVA/Arm and Lm-OVA/Cl-13 mice (CD45.2) on more than 90 days after LCMV infection, and then purified cells containing the same number of K^b^/OVA_257_-specific T cells were transferred into congenic naïve recipients (CD45.1), which were challenged with WT Lm-OVA 1 day later. (D) Survival of mice that were non-transferred with cells (non-transfer) or receiving donor CD8^+^ T cells isolated from Lm-OVA/Arm (Arm) or Lm-OVA/Cl-13 (Cl-13) mice, following Lm-OVA challenge. (E and F) Percentage of donor-derived CD8^+^ T cells (CD45.2) (E), and fraction (F, left) or absolute number (F, right) of donor-derived K^b^/OVA_257_-specific T cells in the spleens and livers of adoptively transferred hosts on day 5 after Lm-OVA challenge.

Protective immunity against Lm re-infection is mediated by CD4^+^ and CD8^+^ Tm cells, with CD8^+^ T cells playing a predominant role [25]. To examine if loss of protective immunity against Lm reinfection in Lm-OVA/Cl-13 mice (Fig 1) is due to impaired memory recall of bacteria-specific Tm cells, we examined recall responses of OVA_257_-specific CD8^+^ and LLO_190_-specific CD4^+^ Tm cells on day 5 following Lm-OVA challenge. In comparison with Lm-OVA/Arm mice, Lm-OVA/Cl-13 mice had much lower percentages of OVA_257_-specific CD8^+^ T cells in their spleens and livers after Lm challenge, as determined by tetramer staining or ICS following stimulation with cognate peptide (Fig 6A and 6B). Similarly, we observed much lower proportions of LLO_190_-specific CD4^+^ T cells in Lm-OVA/Cl-13 than in Lm-OVA/Arm mice by day 5 after Lm challenge (S4 Fig). Together, these data suggest that the recall capacity of both CD4^+^ and CD8^+^ Tm cells specific to Lm was impaired by chronic Cl-13 infection.

To further test whether bystander Tm cells were capable of recalling after removal from the chronically infected hosts, we adoptively transferred an equal number of K^b^/OVA_257_-specific CD8^+^ T cells, which were purified from Lm-OVA/Arm or Lm-OVA/Cl-13 mice (CD45.2) on more than 90 days after LCMV infection, into congenic recipients (CD45.1). One day after transfer, recipient mice were challenged with a lethal dose of Lm-OVA (Fig 6C). The majority of mice (8/9) receiving donor cells from Lm-OVA/Arm mice survived from bacterial challenge. In contrast, most mice receiving donor cells from Lm-OVA/Cl-13 mice succumbed to bacterial challenge on day 5, which were comparable to non-transfer control mice (Fig 6D). Donor T cells and OVA_257_-specific CD8^+^ Tm cells from Lm-OVA/Arm mice expanded and constituted a much higher proportion of total CD8^+^ T cell population in recipients, compared to those from Cl-13 infected mice (Fig 6E and 6F). Together, these data show that the functionality of pre-existing bystander Tm cells is intrinsically impaired by chronic virus infection, resulting in a diminished capacity to mount a strong recall response and confer protective immunity against bacterial reinfection. This impairment persists even after the bystander cells are removed from the chronic viral infection milieu.

## Discussion

The formation of competent immunological memory is critical for rapid control of infections by previously encountered pathogens [4,9]. After maturation, Tm cell maintenance is dependent on environmental signals. During chronic viral infections, persistent antigen exposure can impact the memory repertoire [15]. Numerous data have shown that chronic virus infections negatively affect the development program of virus-specific T cells, which leads to a failure in the formation of functional Tm cells and impaired control of viral infections [10,12,13,26]. However, little is known about how chronic viral infections alter immune responses from the memory pool to unrelated pathogens. Herein, by employing two murine model pathogens, we showed that Lm-OVA immunized mice failed to mount an effective recall response to efficiently eradicate bacteria from a secondary challenge if the host suffered from a chronic virus infection (Fig 1). We found that chronic virus infection not only diminished the pool of pre-existing Lm-OVA specific CD4^+^ and CD8^+^ Tm cells, but also impaired their capacity to produce effector cytokines as well as mount recall responses (Figs 2, 3, 6, and S2–S4). Consistent with our data, Barnstorf *et al*. observed loss of adoptively transferred transgenic OT-I cells and impairment of their functions by chronic infection with a different LCMV strain, docile [17]. Instead of using monoclonal OT-I cells, our experimental system allowed us to examine a natural, polyclonal population of OVA-specific memory T cells induced by a bacterial infection. In contrast to the results from Barnstorf *et al*., we discovered clear phenotypic differences between virus-specific and non-virus-specific memory T cells (see below). More importantly, our study showed for the first time that a chronic infection impaired immunity to another pathogen, resulting in loss of protection against re-infection. These results have important clinical implications, and it would be critical to determine experimentally if our findings are applicable to other chronic infections, particularly in human diseases.

Although both virus- and non-virus-specific T cells become functionally impaired following chronic virus infection, our results showed clear phenotypic differences between these two populations (Figs 2 and 5). Most importantly, non-virus-specific T cells did not show upregulation of inhibitory receptors (Fig 5), a hallmark feature of exhaustion in virus-specific T cells. In addition, we observed differences in kinetics and tissue distribution between the two populations. While virus-specific T cells were rapidly expanding within the first week, we observed substantial loss of non-virus-specific memory T cells during this period in both Arm and Cl-13 infected mice, though the loss is more profound in Cl-13 than Arm-infected mice (Fig 2B). The initial loss of non-virus-specific memory T cells correlated with the timing of active viral replication, and higher viral replication likely contributed to more loss in Cl-13 infected mice (Fig 2B). After the clearance of an acute Arm infection, both virus-specific and non-virus-specific memory T cells remained stable and functional. In chronic Cl-13 infected mice, there was continuous loss in the number and function of non-virus-specific T cells, though much delayed in kinetics compared to virus-specific T cells. Virus-specific T cells were mostly depleted from the peripheral blood and became functionally impaired after 21 days. In contrast, profound loss of non-virus-specific T cells occurred much later (> 60 days, Fig 2), and these cells were still able to produce substantial amounts of cytokines at 40 days after chronic viral infection (Figs 3 and S2). Although virus-specific T cells were undetectable in peripheral blood at late time-points (Fig 2), they were preserved in the spleen and liver (Figs 5 and S1). However, we did not identify an enrichment site for non-virus-specific T cells (Figs 2 and 5).

The mechanisms of how non-virus-specific memory T cells are affected by a chronic viral infection are not known. The phenotypic and kinetic differences we observed between virus-specific and non-virus-specific T cells suggest that there are likely differences in the underlying mechanisms. In the canonical model proposed from existing data, T cell exhaustion is generally triggered by three signals: 1) persistent antigenic stimulation, 2) sustained co-inhibitory signals transduced by immune receptors, and 3) profound inflammation within the chronic infection environment [16,27]. Virus-specific T cells receive cumulative signals from TCR, inhibitory receptors (like PD-1), and inflammatory cytokines. In contrast, non-virus-specific T cells likely do not receive all three signals. First, antigenic stimulation is unlikely involved in functional defects of non-virus-specific memory T cells, since LCMV does not have epitopes that are cross-reactive with OVA-specific T cells. Second, sustained inhibitory signals may only play a minor role in functional defect of non-virus-specific T cells. Barnstorf *et al*. reported less than 1/3 of memory OT-I CD8^+^ T cells in chronic virus infected host express high levels of PD-1 [17]. We did not observe elevated expression of PD-1, TIM-3 and LAG-3 on OVA-specific T cells at any time-points during chronic virus infection (Fig 5). Consistent with the “Signal Strength” model [16], lack of antigenic stimulation and weak inhibitory signals may explain delayed onset of functional defects in non-virus-specific T cells, compared to virus-specific T cells.

Non-virus-specific T cells may experience the same profound inflammatory environment as virus-specific T cells in chronically infected hosts [16,27]. In support of a role for inflammatory signals, loss of non-virus-specific memory T cells and their functions correlates with timing and levels of active viral replication that drives inflammatory responses. Inflammatory cytokines such as type I IFNs (IFN-α/β) and IL-12 are the major sources of signal 3 required for T cell responses [28]. Type I IFNs and IL-12 are known to promote the generation of terminally differentiated T cells (KLRG1^+^CD127^lo^) [29,30]. Type I IFNs have also been reported to induce apoptosis of memory phenotype T cells (CD44^hi^) [31]. In addition, Barnstorf *et al*. observed sustained IL-6 production during chronic LCMV infection; IL-6 induces terminal differentiation on bystander memory OT-I cells [17]. These inflammatory cytokines together may play critical roles in the numeric loss and phenotypical alterations of non-virus-specific memory T cells. Interestingly, neither blockade of IFNAR nor IL-6R is sufficient to fully restore the number and function of bystander memory CD8^+^ T cells [16,17], suggesting the involvement of additional factors in driving bystander memory T cell dysfunction. These factors could include: 1) dysregulated expression of critical survival factors (e.g. IL-7, IL-15 for homeostatic proliferation/maintenance) [11], and 2) skewed differentiation of immunosuppressive dendritic cells (DCs) by sustained inflammatory signals [32]. Further studies are needed to investigate the role of these factors and fully understand the underlying mechanisms driving functional defects of non-virus-specific T cells during chronic viral infection.

Non-virus-specific Tm cells from chronic virus infected hosts were not only defective in effector functions, but also incapable of mounting an effective recall response (Figs 3, 6, and S2–S4). Similarly, Barnstorf *et al*. showed that memory OT-I CD8^+^ T cells cannot mount a robust secondary expansion in chronic LCMV infected mice [17]. The reasons for impaired secondary expansion are not known and could be due to limited expression of IL-2Ra following re-encounter of antigen [33], lack of DCs for efficient antigen presentation [32,34], or loss of response to inflammatory cytokines [35]. Furthermore, our results showed that non-virus-specific T cells from chronical Cl-13 infected mice were defective in recall responses even after transferred into new naïve recipients and were unresponsive to even PMA/I stimulation which bypasses proximal TCR signaling. (Figs 4 and 6). These results indicate that a chronic viral infection causes intrinsic defects in pre-existing bystander Tm cells specific to other pathogens (Fig 6). We and others have previously shown that epigenetic remodeling plays a critical role in transcriptional activation of the key effector genes and functional impairment during T cell exhaustion [36–43]. It remains to be defined how a chronic viral infection alters the transcriptional circuits and epigenetic landscapes of bystander Tm cells. Investigation into these questions will likely identify druggable targets to restore the function of compromised non-virus-specific T cells.

In summary, our study shows that bystander chronic viral infections are detrimental to the maintenance of long-lasting protective immunity against previously encountered pathogens. The results call for further investigations into broad impact that chronic exposure to inflammatory signals has on the immune system. Understanding the underlying mechanisms will help develop novel therapies that prevent the exhaustion of virus-specific T cells, while retaining the immune memory and competence to control other pathogens.

## Materials and methods

### Mice and ethics statement

Six-week-old female C57BL/6 (CD45.2^+^ or CD45.1^+^) mice were purchased from the National Cancer Institute (Bethesda, MD). Mice were maintained under specific pathogen-free conditions at animal facilities of the University of Pennsylvania, and were used in accordance with the Animal Care Guidelines of the University of Pennsylvania. Animal experiments were approved by the University of Pennsylvania Institutional Animal Care and Use Committee (IACUC, protocol # 806299).

### Infections

Mice were immunized intravenously (i.v.) with Lm-OVA, as described previously [29]. More than 30 days after Lm-OVA infection, mice were infected with either LCMV Arm or Cl-13 strain, as described [23,44]. CD4^+^ T cells were depleted by intraperitoneal injection of 200 μg anti-CD4 antibody (GK1.5, BioXcell) at 1 day prior and post LCMV infection [44]. For rechallenge experiments, mice were infected i.v. with 1×10^6^ CFUs of WT Lm-OVA.

### Flow cytometry

Surface staining and intracellular cytokine staining (ICS) were described previously [42]. Briefly, single-cell suspensions from peripheral blood, spleen or liver were stained with LIVE/DEAD Fixable Aqua Dye (Invitrogen) to exclude dead cells, then incubated with surface antibodies (eBiosciences) in combination with tetramers (NIH Tetramer Core Facility, Atlanta, GA). For ICS, cells were first cultured at 37°C for 5 hours in the complete medium (RPMI 1640 containing 10% FBS, 1% HEPES, 1% non-essential amino acid, 2 mM L-glutamine, 50 μM β-mercaptoethanol, 100 U/ mL penicillin and 100 μg/mL streptomycin; Gibco), in the presence of LLO_190-201_, GP_33-41_ or OVA_257-264_ peptides (1 μg/mL), or PMA (50 ng/mL) + Ionomycin (0.5 μg/mL) (Sigma Aldrich). Brefeldin A and monensin were added to the culture for the last 4 hours. Cells were stained for viability and surface antigens as described above, followed by permeabilization (BD Cytofix/Perm) for intracellular staining. Samples were acquired on LSRII flow cytometer (BD Biosciences) and data were analyzed with FlowJo software (Treestar). Details about the reagents and softwares we used are shown in S1 Table.

### Adoptive cell transfer

CD8^+^ T cells were purified from splenocytes by magnetic cell sorting (Miltenyi Biotec.), stained with K^b^/OVA_257_ tetramer to determine the number of OVA-specific cells. 5000 OVA-specific CD8^+^ T cells were transferred i.v. to recipient mice, which were challenged with Lm-OVA 1 day later as described previously [29].

### Statistical analysis

Statistical analyses were described previously [45]. Briefly, the data were first evaluated for normality by the Shapiro-Wilk normality test. If the data passed normality test, we performed statistical analysis with unpaired Student’ s t-test, or one-way ANOVA with subsequent Tukey’s post-tests; if not, data were analyzed by the Mann-Whitney U test, or Kruskal-Wallis test followed by Dunns multiple-comparison test. Statistical significance was defined as follows: * *p* < 0.05, **p < 0.01, and ***p < 0.001.

## Supporting information

S1 Data Excel spreadsheets contain the underlying numerical data for Figs 1B, 1C, 2B, 2C, 3A, 3B, 3C, 3D, 3E, 3F, 3G, 4B, 4D, 6A, 6B, 6D, 6E, 6F, S2B, S2D, S3, S4A and S4B. (XLSX)

S1 Table Details about the reagents and software we used. (XLSX)

S1 Fig Virus-specific T cells become exhausted following chronic virus infection in Lm-OVA immunized mice. (A) Experimental design. Mice were infected with Δ*actA* Lm-OVA, and more than 30 days after Lm-OVA infection, mice were infected with LCMV Arm or Cl-13. Virus-specific T cells were detected at day 45 after LCMV infection. (B) Expression of CD44, CD127, PD-1, TIM-3 and LAG-3 on D^b^/GP_33_^+^ CD8^+^ T cells in the spleens of Lm-OVA/Arm or Lm-OVA/Cl-13 mice. (C) Expression of CD107a and production of IFN-γ, TNF-α and IL-2 by CD8^+^ T cells from the spleens of Lm-OVA/Arm or Lm-OVA/Cl-13 mice after GP_33_ peptide stimulation.
(TIF)

S2 Fig Chronic virus infection leads to a progressive loss of Lm-OVA-specific Tm cells. Infection of mice was the same as described in Fig 2. (A-D) Peripheral mononuclear cells isolated from Lm-OVA/Arm and Lm-OVA/Cl-13 mice at various time points post LCMV infection were stimulated with OVA_257_ (A & B) or LLO_190_ (C & D) peptides, respectively. Dynamic changes in the percentages of CD8^+^ (A & B) and CD4^+^ T cells (C & D) producing IFN-γ after peptide stimulation were shown. Asterisks indicate the statistical significance between Arm and Cl-13 mice (*, p<0.05; **, p<0.01; ***, p<0.001).
(TIF)

S3 Fig Lm-OVA-specific CD4^+^ Tm cells lose polyfunctionality after chronic virus infection. Infection of mice was described in Fig 2. More than 90 days after LCMV infection, CD4^+^ T cells in the spleen of Lm-OVA/Arm or Lm-OVA/Cl-13 mice were analyzed for their co-production of IFN-γ, TNF-α and IL-2, in the presence of LLO_190_ peptide stimulation. Cytokines: 1, IFN-γ^+^TNF-α^-^IL-2^-^; 2, IFN-γ^+^TNF-α^+^IL-2^-^ and IFN-γ^+^TNF-α^-^IL-2^+^; 3, IFN-γ^+^TNF-α^+^IL-2^+^.
(TIF)

S4 Fig Lm-OVA-specific CD4^+^ Tm cells display impaired recall in chronic virus infected host. Infection and challenge of mice were the same as described in Fig 1. **(A & B)** Recall responses of I-A^b^/LLO_190_-specific CD4^+^ T cells in the spleen and liver of Lm-OVA/Arm and Lm-OVA/Cl-13 mice were analyzed by MHC Class II tetramer staining (A) or ICS for IFN-γ after LLO_190_ peptide stimulation (B).
(TIF)
