# Supplementary figures and images for: Chronic viral infection impairs immune memory to a different pathogen
Source: PLoS Pathog. 2024 Mar 28;20(3):e1012113. doi: 10.1371/journal.ppat.1012113 (PMC11003680; doi:10.1371/journal.ppat.1012113)

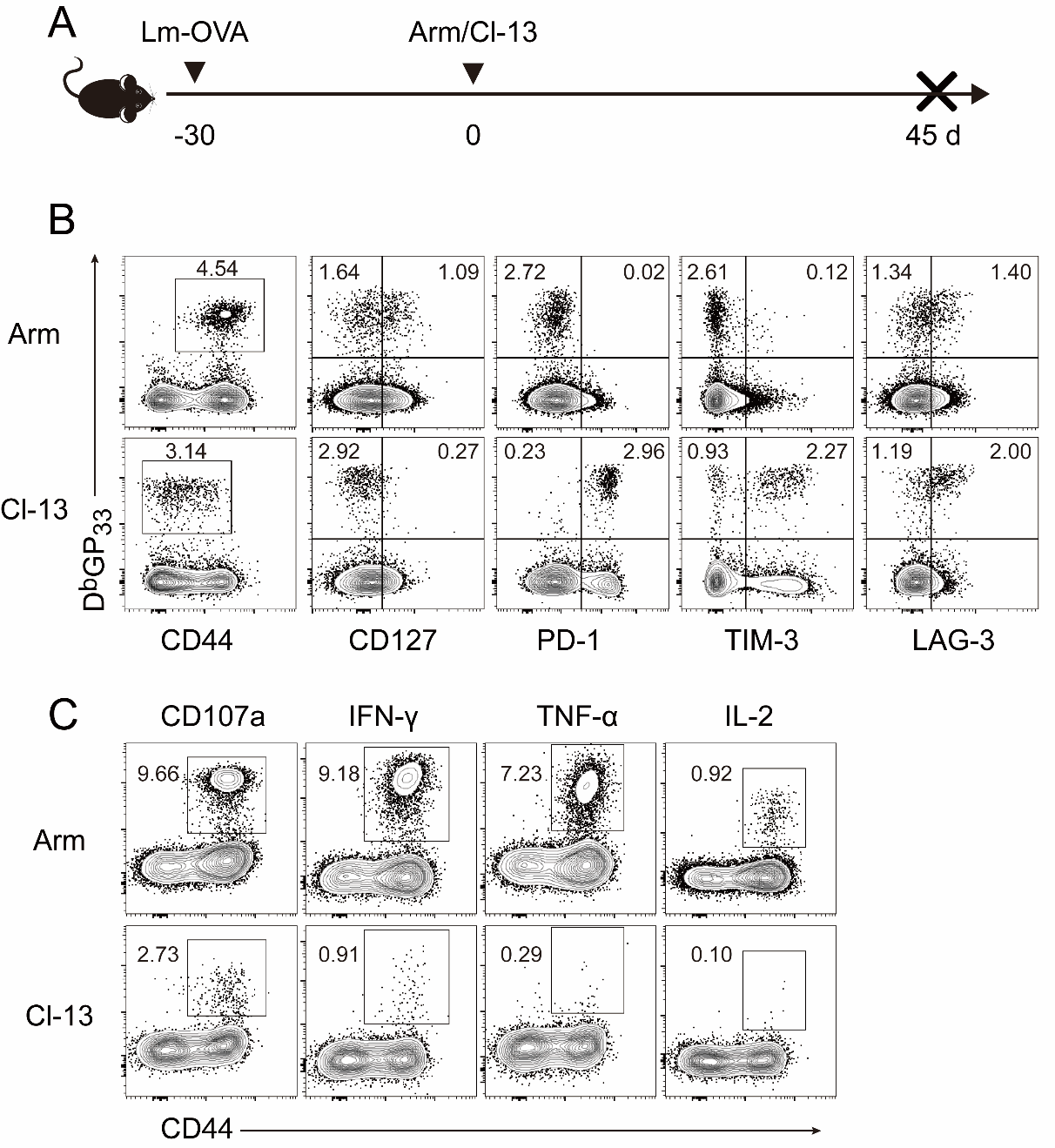

Supplement: S1 Fig — (A) Experimental design. Mice were infected with ΔactA Lm-OVA, and more than 30 days after Lm-OVA infection, mice were infected with LCMV Arm or Cl-13. Virus-specific T cells were detected at day 45 after LCMV infection. (B) Expression of CD44, CD127, PD-1, TIM-3 and LAG-3 on Db/GP33+ CD8+ T cells in the spleens of Lm-OVA/Arm or Lm-OVA/Cl-13 mice. (C) Expression of CD107a and production of IFN-γ, TNF-α and IL-2 by CD8+ T cells from the spleens of Lm-OVA/Arm or Lm-OVA/Cl-13 mice after GP33 peptide stimulation. (TIF) [file ppat.1012113.s003.tif]

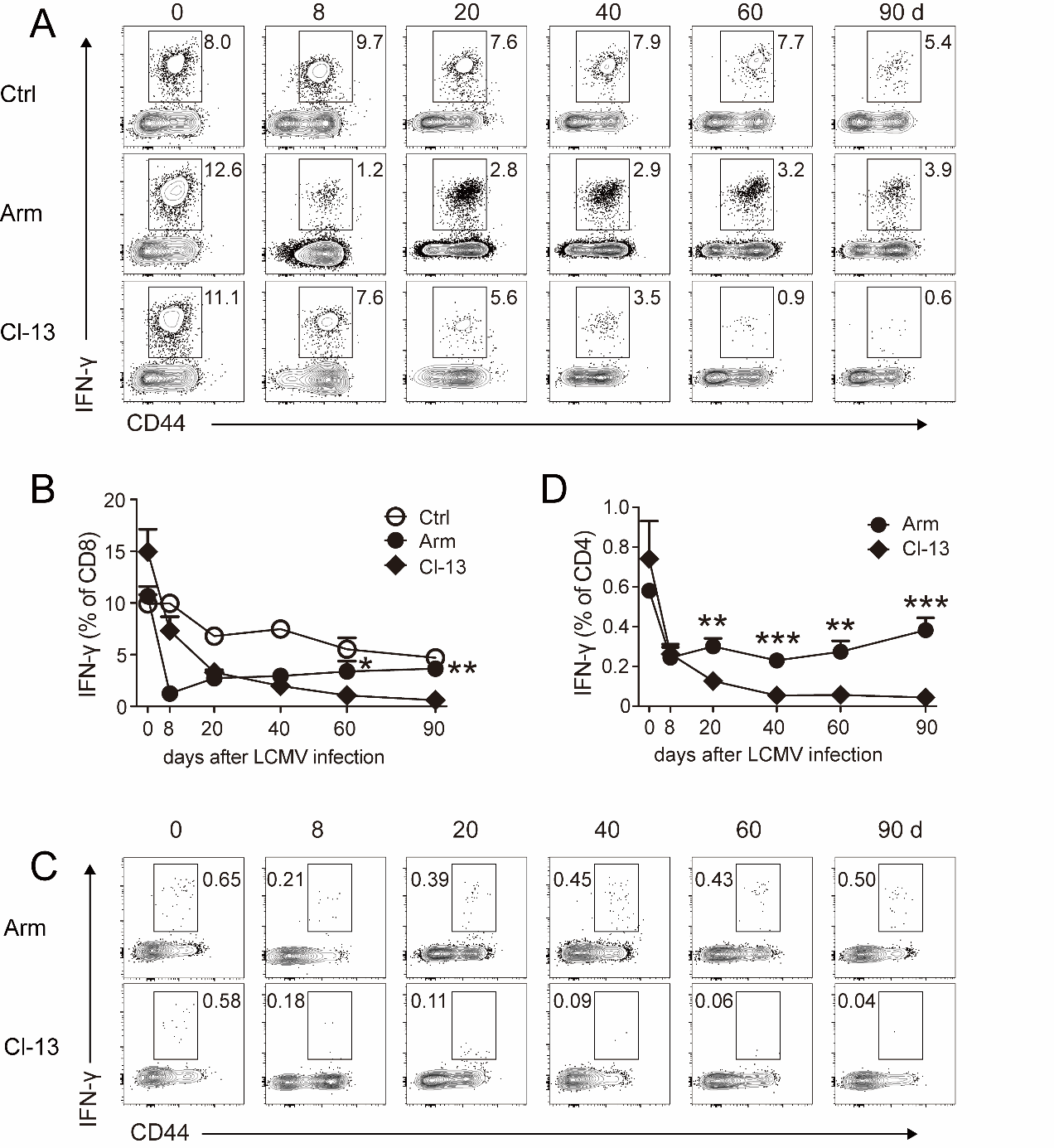

Supplement: S2 Fig — Infection of mice was the same as described in Fig 2. (A-D) Peripheral mononuclear cells isolated from Lm-OVA/Arm and Lm-OVA/Cl-13 mice at various time points post LCMV infection were stimulated with OVA257 (A & B) or LLO190 (C & D) peptides, respectively. Dynamic changes in the percentages of CD8+ (A & B) and CD4+ T cells (C & D) producing IFN-γ after peptide stimulation were shown. Asterisks indicate the statistical significance between Arm and Cl-13 mice (*, p<0.05; **, p<0.01; ***, p<0.001). (TIF) [file ppat.1012113.s004.tif]

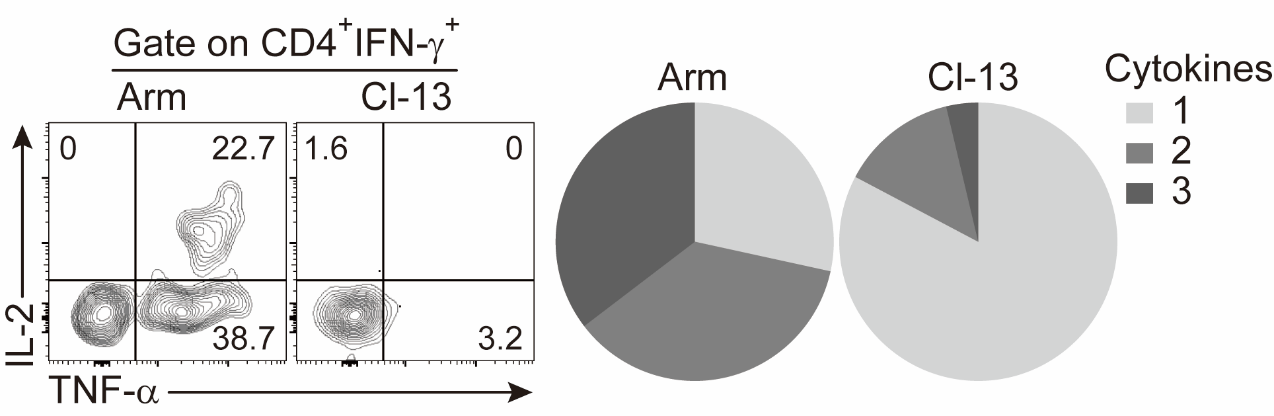

Supplement: S3 Fig — Infection of mice was described in Fig 2. More than 90 days after LCMV infection, CD4+ T cells in the spleen of Lm-OVA/Arm or Lm-OVA/Cl-13 mice were analyzed for their co-production of IFN-γ, TNF-α and IL-2, in the presence of LLO190 peptide stimulation. Cytokines: 1, IFN-γ+TNF-α-IL-2-; 2, IFN-γ+TNF-α+IL-2- and IFN-γ+TNF-α-IL-2+; 3, IFN-γ+TNF-α+IL-2+. (TIF) [file ppat.1012113.s005.tif]

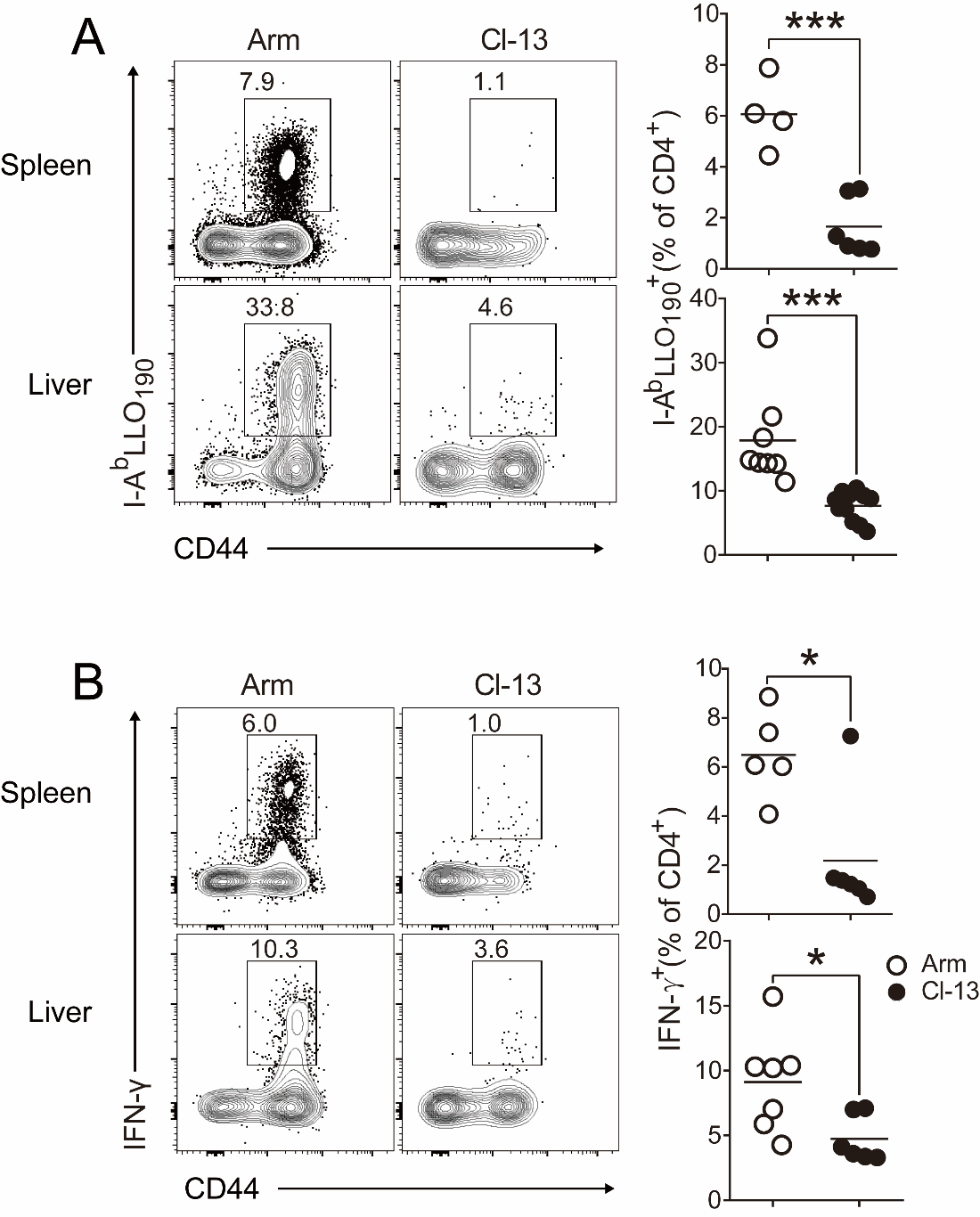

Supplement: S4 Fig — Infection and challenge of mice were the same as described in Fig 1. (A & B) Recall responses of I-Ab/LLO190-specific CD4+ T cells in the spleen and liver of Lm-OVA/Arm and Lm-OVA/Cl-13 mice were analyzed by MHC Class II tetramer staining (A) or ICS for IFN-γ after LLO190 peptide stimulation (B). (TIF) [file ppat.1012113.s006.tif]
